# Influence of Mining and Vegetation Restoration on Soil Properties in the Eastern Margin of the Qinghai-Tibet Plateau

**DOI:** 10.3390/ijerph17124288

**Published:** 2020-06-16

**Authors:** Yunlong Hu, Zhifeng Yu, Xiangling Fang, Weixiong Zhang, Jinrong Liu, Feng Zhao

**Affiliations:** 1 State Key Laboratory of Grassland Agro-Ecosystems, College of Pastoral Agriculture Science and Technology, Lanzhou University, Lanzhou 730030, China; huyl17@lzu.edu.cn (Y.H.); xfl2807@163.com (X.F.); 2 Third Institute Geological and Mineral Exploration of Gansu Provincial Bureau of Geology and Mineral Resources, Lanzhou 730030, China; yuzhifeng72@163.com (Z.Y.); yyvisume@163.com (W.Z.); Zhaof@163.com (F.Z.)

**Keywords:** Qinghai-Tibet plateau, mine restoration, soil nutrients, soil enzyme activity, trace metals

## Abstract

Mining causes serious destruction of the surface morphology and soil structure of lands, and vegetation restoration on post-mining lands provides an effective way for soil and water conservation. To determine the influence of mining and vegetation restoration on soil properties in the eastern margin of the Qinghai-Tibet Plateau, four land sites, including two vegetation restoration sites (restorated by *Elymus nutans* and *Picea crassifolia*, respectively), one non-vegetated mining site and one native grassland site, were selected. Fifty-two topsoil (0–10) samples were collected from these four sites, and then soil properties, trace metals and soil enzyme activities were analyzed. The results showed that there was an increase in soil pH (>8.0) after mining, while vegetation restoration decreased the soil pH compared with native grassland; the soil organic matter and total nitrogen in the site restored with *E. nutans* increased by 48.8% and 25.17%, respectively, compared with the site restored with *P. crassifolia*. The soil enzyme activities decreased after mining, and there were no significant increases in urease, phosphatase, β-glucosidase and β-1,4-*N*-acetylglucosaminidase activities after five years of restoration. In addition, the contents of soil trace metals (cadmium, chromium, mercury, lead and zinc) after mining were lower than the Chinese threshold (GB 15618/2018), but the content of arsenic in non-vegetated soil and *P. crassifolia*-restored soil exceeded the threshold by 22.61 times and 22.86 times, respectively. Therefore, As-contaminated land areas should be accurately determined and treated in a timely way to prevent arsenic from spreading, and plant species with tolerance to alkaline soil should be selected for vegetation restoration on post-mining lands.

## 1. Introduction 

Over the past few decades, human activities have been the most direct factors causing changes to the Earth’s surface and ecosystems [1]. Mining activities, as with most activities to sustain human livelihood, have resulted in the most intense impact on the ecosystem structure and function of mining areas [2,3]. Severe environmental damage and ecological degradation, such as removal of natural vegetation, soil erosion and quality decline, are very common in mining areas [4]. Vegetation restoration projects, including tree planting, agricultural reclamation and other ventures, which could accelerate the natural restoration processes of soil and enhance biological diversity of land degradation by mined activities, plays a significant role in enhancing soil health and mitigating soil erosion [5,6].

Soil properties, include soil organic matter, total nutrient element concentration, available nutrient element concentration, pH, and electrical conductivity, etc., have been identified as indicators of soil health [7]. Soil health is an important component in the restoration of ecosystems due to its physical, chemical and biological (nutrient) support for plant recolonization and establishment [8]. Vegetation restoration could enhance the accumulation of soil organic matter and nutrients in the soil and these processes improve the soil conditions for subsequent species colonization and ecosystem development [9]. Therefore, thoroughly knowledge of the likely changes in soil organic matter content and the proportion of nutrients in the soil during the initial restoration period is essential for human beings to predict vegetation restoration status and soil conditions. 

Trace elements are typically characterized by very low concentrations in the environment (below 0.1% in natural media; below 0.01% in plant or animal tissues) [10]. Trace elements include various chemical families—metals (e.g., lead (Pb), cadmium (Cd), chromium (Cr), and zinc (Zn)) and metalloids (arsenic (As) and mercury (Hg)) [11]. Most trace metals pose serious environmental and health risks because they are toxic and could become labile when the normal threshold is exceeded and they are exposed to the environment [12]. Both heavy metals and metalloids are contaminants in mining and smelting areas as they accumulate in soils, plants, and water streams, posing serious threats to the ecosystem health and nearby habitats, including fauna, flora, and microfauna [13,14,15]. Most of these effects persist over large expanses of land, even long after mining activities have ceased [2,16]. However, trace metals in soils can be derived from either parent materials and bedrock or anthropogenic sources [17]. Therefore, investigating the content of trace metals in mining soils can help us not only to understand the quality of the soils, but also to distinguish between the geogenic or anthropogenic sources of these trace metals, and finally, control the diffusion of trace metals pollution in a timely fashion. 

Soil enzymes, produced by both plants and soil microorganisms, act as the primary mediators of soil biological processes. Enzymes catalyze important transformations in nutrient cycles, including those of carbon, nitrogen, and phosphorus [18], playing an important role in maintaining soil ecology, physicochemical properties, soil fertility and soil health [19,20]. Soil enzymes also have high sensitivity to the changes caused by both natural and anthropogenic factors [21]. Therefore, soil enzymes have been suggested as suitable indicators of soil quality because they are strictly related to the nutrient cycles and rapid response of soil microenvironments and properties. Among the different enzymes in soils, urease and β-glucosidase (BG), β-*N*-acetylglucosaminidase (NAG) and phosphatases are involved in C, N, and P transformation, respectively [22]. 

The Qinghai-Tibet Plateau is the highest (averaging about 4500 m) and largest (2.5  ×  10^6^ km^2^) plateau on Earth [23,24]. The northeastern margin is at the junction of the southeast and southwest warm moist air, and is also the junction of the climate ecology of the Qinghai-Tibet Plateau and the Loess Plateau with altitude 1500–4200 m and mean annual precipitation ranges between 600 and 700 mm. It is an important water source recharge area in the upstream headwater regions of the Yellow River. Due to its special geographical location, the eco-environment of this region is particularly sensitive to climate change and anthropogenic activities. Previous research on this region was dominantly focused on climate change and grazing, with less research attention being paid to mining activities. 

It is clear that an improved understanding of soil nutrients, trace metal contamination and enzyme activity in soils is crucial to the sustainable development of the mining area ecosystem. In this study, three sites restorated by *Picea crassifolia* (RPc), *Elymus nutans* (REn) and non-vegetated (NV), respectively, and a native grassland (NG) in the northeastern edge of Qinghai-Tibet Plateau were selected. *E. nutans* is a native grass species commonly used to build artificial grasslands and reseeding in degraded alpine meadows in this region [25], and *P. crassifolia* is a unique tree species on the northeastern edge of the Qinghai-Tibet Plateau [26]. For the reason that, these two types of plant species become popular vegetation restoration plants in this area. The research objectives were to: (1) determine soil nutrients dynamics after mining and vegetation restoration; (2) identify the distribution of trace metals in soils of the mining area; (3) assess the effects of vegetation cover on soil functionality in restored areas. This study will improve the understanding of soil statues in the Qinghai-Tibet Plateau area and the effect of different types of monoculture restoration. 

## 2. Materials and Methods

### 2.1. Study Sites

**Figure 1:**
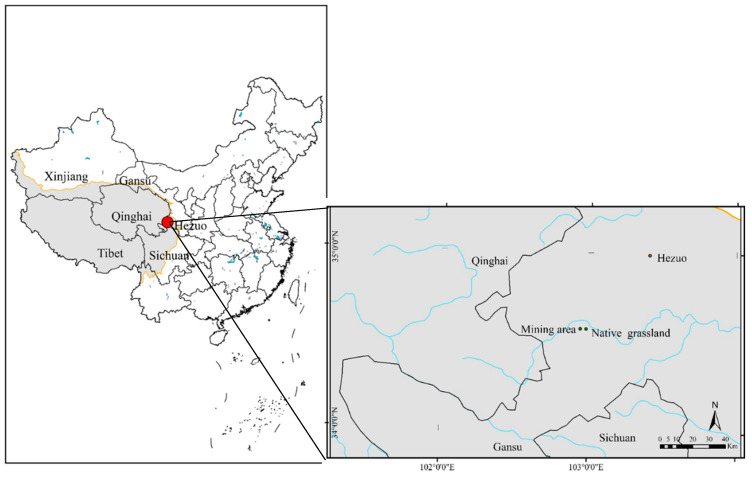
Location of the study area.

The study area is situated on the northeastern edge of the Qinghai-Tibet Plateau (34°57′ N/102°44′ E for native grassland and 34°57′ W/102°48′ N for mining area (Figure 1)). The altitude of this area ranges from 3100 to 3500 m, and the climate is cold and humid-alpine, with a mean annual rainfall of 450–780 mm. The mean annual temperature is 1.2 °C, varying from an average of −10.7 °C in January to 11.7 °C in July, with an average of 270 frost days per year. The main vegetation types in the area are alpine shrubs and meadows, and the soil types are dominated by meadow soils, chernozems and chestnut soil.

Mine exploitation of this area began in 1994, and the most prevalent mining technique changed from open-pit to underground mining in 2003. The two restored plots selected for the study were adjacent to each other and were previously open-pit mining sites. Before mining, the surface soil layer is stockpiled to the surrounding open space. Before restoration, the waste rock is used to fill the pit, and then the stockpiled topsoil was used to overlay reconstructed landscapes during revegetation to form a topsoil layer of about 30 cm in depth. The surfaces of both restored sites were leveled by earth-moving machinery before planting, which occurred in June 2013. The non-vegetated site was located in an area that has just experienced excavation, one kilometer away from the two restored sites, with no vegetation cover, also leveled four months prior when soil sampling. The natural grassland plot is 15 km away from the mining area, with the same slope and altitude as the restored sites. The restoration area of the herb plants was 4270 m^2^, which was recovered by hand broadcast seeding with *E. nutans* at a rate of 50 kg seeds over the whole area; the area restored with woody plants was 7090 m^2^, using *P. crassifolia* at a spacing of 2 m × 2 m. 

### 2.2. Soil Sample Collection 

According to the grid distribution point method, 52 topsoil samples (0–10 cm) were collected across the study area in July 2018. Five sub-samples were selected within a 3 m × 3 m square and well mixed into a composite sample (approximately 500 g). The coordinates of the sampling points were recorded in a GPS receiver. Plant fragments and visible rock fragments larger than 2 mm were removed by hand. Soil samples were divided into two equal parts for further analysis, one dried at room temperature for soil nutrient and trace elements analysis, and the other stored at −20 °C for soil enzyme analysis.

### 2.3. Soil Chemical Analyses

Soil pH and conductivity was measured using a suspension made of 5 g of air-dried sieved soil in 25 mL of deionized water (1:5 sample to deionized water ratio). SOM was measured by the K_2_Cr_2_O_7_ oxidation—external heating method [27]. Total N and available N were determined using a continuous flow analyzer (SEAL, Norderstedt, Germany). Total P and available soil P were measured by the Mo–Sb colorimetric method following H_2_SO_4_-HClO_4_ digestion, and NaHCO_3_ was the extractant. Total K and available K were measured using flame emission spectroscopy after HF-HClO_4_ digestion, and the extractant was ammonium acetate [28]. Total Sulphur (S) was measured by EDTA indirect titration method [27]. For Hg determination [29], 0.5 g of each soil sample was extracted use 10 mL aqua regia (2 mol L^−1^ HNO_3_ and 4 mol L^−1^ HCl) at 100 °C for 2 h in Teflon digestion vessel. Hg concentrations were measured by cold vapor-atomic absorption spectrometry (CV-AAS) (Perkin-Elmer, Waltham, MA, USA) following a reduction with SnCl_2_. 

The concentrations of soil metals (Cr, Zn, As, Cd and Pb) were determined by aqua regia digestion method [30,31]. In brief, 0.5 g dry soil sample was weighed in a Teflon digestion vessel with 12 mL aqua regia (a mixture of 68% HNO_3_ and 38% HCl at 1:3 *v*/*v*) until reduced to 5 mL. Samples were filtered and diluted with deionized water to 50 mL for analysis. The total metal concentrations in the extracts were determined using an inductively coupled plasma mass spectrometer (ICP-MS, Agilent, Santa Clara, CA, USA), total content of trace metals was expressed in mg/kg dry soil. In this study, the standard soil reference material GBW 07425 (China soil standard material) was used for quality control. Three blank controls and standard materials were set for each batch of samples. The measured values for reference samples were between 92% and 108% of the certified values for all five elements, and measurement errors were less than 10%.

### 2.4. Soil Enzyme Activity Analysis

Four soil enzymes involved in cycles of C, N, and P were assessed. For urease, 5 g of the moist soils were incubated with 10 mL of 10% urea solution and 20 mL citric acid-sodium buffer solution at 37 °C for 24 h, and the urease activity expresses as mg NH_3_-N per gram of soil [18,32]. Phosphatase activity was assayed using a disodium phenyl phosphate method. 5 g of the moist soils were incubated with 2.5 mL toluene and 20 mL 0.5% buffered disodium phenyl phosphate solution at 37 °C for 24 h, and the unit was expressed as mg phenol per gram of soil [32]. For the determination of NAG activity, 1 g of the moist soils were incubated with 4 mL of 100 mM acetate buffer solution and 1 mL of 10 mM *p*-nitrophenyl-*N*-acetyl-β-d-glucosaminide solution at 37 °C for 24 h, and the unit was expressed as μg *p*-nitrophenol per gram of soil. For BG activity, 1 g of the moist soils were incubated with 4 mL of MUB buffer solution, 0.25 mL toluene and 10 mL of 0.05 mM *p*-nitrophenyl-l-β-d-glucopyranoside solution at 37 °C for 24 h, and the unit was expressed as μg *p*-nitrophenol per gram of soil [33,34].

### 2.5. Statistical Analysis

Differences in soil indicators among different soil sites were tested using one-way analysis of variance (ANOVA) and comparisons between means were performed with the Tukey’s HSD (honestly significant difference) test (*p* = 0.05). Principal component analysis (PCA) was used to assess differences and clusters in soil variables between soil materials. All analyses were performed with *R* statistical software version 3.6.0. The *R* package FactoMineR and factoextra were used for Visualization and Interpretation of the PCA analysis. The bar charts were drawn using OriginPro 8.6 (OriginLab, Northampton, MA, USA). 

## 3. Results

### 3.1. Soil Properties

In this study, the native grassland soil pH f was moderately alkaline (7.36). After the soils were disturbed by mining activities, the pH clearly increased. The highest pH was observed in NV (8.61), and pH for RPc and REn site was 8.58 and 8.26, respectively (Table 1). The highest EC value also appeared on NV (260.47 mS m^−1^), the EC value of RPc (208.60 mS m^−1^) and REn (170.90 mS m^−1^) is relatively lower, and EC value was not significantly different among the three sites with vegetation cover (Table 1).

The content of SOM and TN decreased significantly in the disturbed soil. Both the SOM (94.46 g kg^−1^) and TN (3.38 g kg^−1^) content in REn sites were significantly greater than at the RPc site (16.85 g kg^−1^ for SOM and 1.10 g kg^−1^ for TN) (*p* < 0.05), but there is no significant difference between RPc and NV (Table 1). The highest value of TP (1.03 g kg^−1^) was recorded at the REn sites, significantly higher than the RPc, NG and NV, but there is no significant difference between RPc (0.81 g kg^−1^) NG (0.85 g kg^−1^) and NV (0.80 g kg^−1^) (*p* > 0.05). Both the TK content in RPc (22.04 g kg^−1^) and REn (22.65 g kg^−1^) are significantly greater than NG (20.29 g kg^−1^), as well as significantly greater than NV (19.68 g kg^−1^). The S content of NV with 0.167% was 2.9 times, 7.3 times, and 9.3 times higher than in native grassland, REn sites and RPc sites, respectively (Table 1).

The available nutrients content was significantly different between native grassland and disturbed soils (Table 1). For AN, it was 283.85 mg kg^−1^ in NG, which was significantly greater than that in RPc (106.51 mg kg^−1^), REn (115.55 mg kg^−1^) and NV (78.61 mg kg^−1^), while there was no significant different between these disturbed sites (*p* > 0.05). For AP, it was 304.2 mg kg^−1^, 266.1 mg kg^−1^, 324.3 mg kg^−1^ in REn, RPc and NE, respectively, while highest value was 464.8 mg kg^−1^ in NV. The AK content in NG (28.85 mg kg^−1^) and REn (26.51 mg kg^−1^) was significantly greater than RPc (6.65 mg kg^−1^) and NV (7.31 mg kg^−1^).

### 3.2. Cotent of Trace Metals and CN in Soils

**Figure 2:**
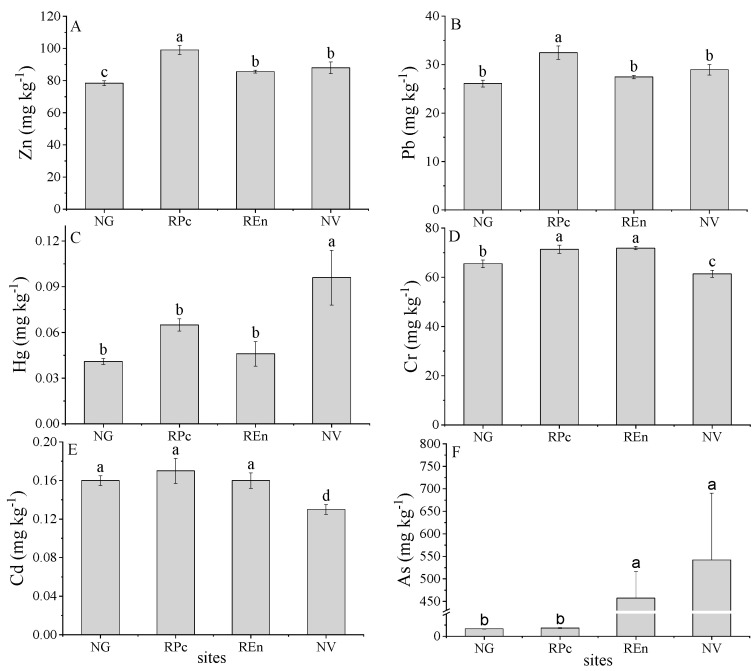
Concentrations of zinc (Zn) (**A**), lead (Pb) (**B**), mercury (Hg) (**C**), chromium (Cr) (**D**), cadmium (Cd) (**E**), arsenic (As) (**F**) in four sites. Different lowercase letters (a, b, c, d) indicate a significant difference under different sites based on ANOVA (*p* < 0.05). NG = native grassland, RPc = restoration by *Picea crassifolia*, REn = restoration by *Elymus nutans*, NV = non-vegetated.

In this study, both of the native grass land and previous studies conducted in this region are set as background values [35,36]. The trace metals content of native grassland was As (16.5 mg kg^−1^), Cd (0.16 mg kg^−1^), Cr (65.47 mg kg^−1^), Hg (0.04 mg kg^−1^), Pb (26.1 mg kg^−1^), Zn (78.45 mg kg^−1^), and CN (0.088 mg kg^−1^) (Figure 2, Table 1). The trace metals content in NG was roughly similar to that of previous studies [35]. Except for Cd, other trace metals in disturbed sites are greater than the background value and NG. The highest value of Cd, Pb, Zn are from REn (Figure 2A,B,D,E). The Hg content of REn, RPc and NV was 43%, 60%, and 73% higher than the background values, respectively (Figure 2C).The average As content value in the RPc and NV was 33 and 39 times greater than the background value, respectively, and As content in REn sites was 18.23 mg/kg, which exceeded the background value by 23% (Figure 2F). For CN, it was 0.088 mg kg^−1^ in NG, which was significantly greater than that in RPc (0.027 mg kg^−1^), REn (0.050 mg kg^−1^), and NV (0.56 mg kg^−1^) (Table 1). 

### 3.3. Soil Enzyme Activities

**Figure 3:**
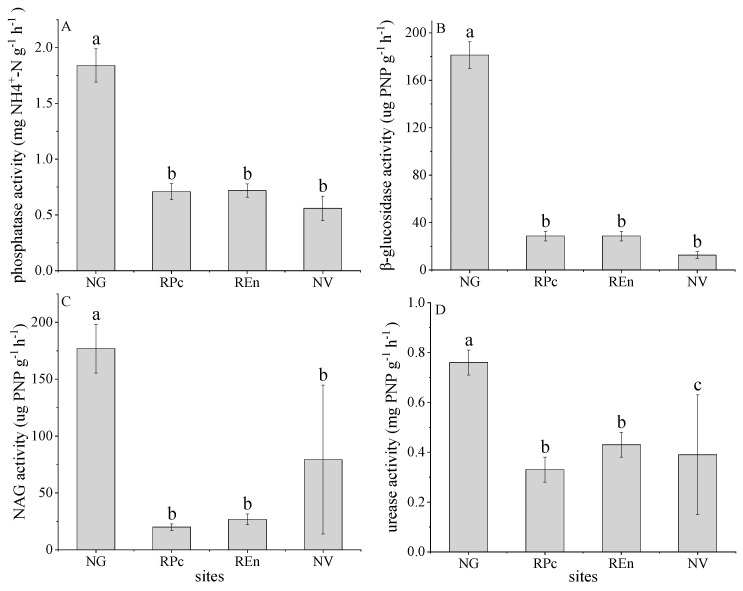
Phosphatase activities (**A**), β-glucosidase activities (**B**), β-1,4-*N*-acetylglucosaminidase activities (NAG) (**C**), urease activities (**D**) in four sites. Different lowercase letters (a, b, c) indicate a significant difference under different restoration sites based on ANOVA (*p* < 0.05).

Soil enzyme activity decreased significantly in disturbed soils (*p* < 0.05). Compared with grassland, the phosphatase activity in RPc and REn top soils decreased by 0.71 mg PNP g^−1^ h^−1^ (38.58%) and 0.72 mg PNP g^−1^ h^−1^ (39.13%), respectively; compared with NV (0.56 mg PNP g^−1^ h^−1^), although there are no significant differences in the data, the phosphatase activity of RPc and REn increased by 22.22% and 21.12%, respectively (Figure 3A). For β-glucosidase, which activity changes similar to phosphatase, the β-glucosidase activity in RPc and REn decreased by 28.68 μg PNP g^−1^ h^−1^ and 28.66 μg PNP g^−1^ h^−1^, respectively; while increased by 56.10% and 56.07% for RPc and REn, respectively (Figure 3B). The NAG activity in RPc and REn top soils decreased by 19.95 μg PNP g^−1^ h^−1^ (11.28%) and 26.73 μg PNP g^−1^ h^−1^ (15.10%), respectively; however, the average content of NAG activity in NV soils was greater than RPc and REn (Figure 3C). Compared with grassland, the urease activity in RPc and REn top soils decreased by 0.33 mg PNP g^−1^ h^−1^ and 0.43 mg PNP g^−1^ h^−1^, respectively, and which activity in NV (0.39 mg PNP g^−1^ h^−1^) significant lower than RPc and REn (Figure 3D). 

### 3.4. Relationships between Soil Properties, Trace Metals and Enzyme Activities

**Figure 4:**
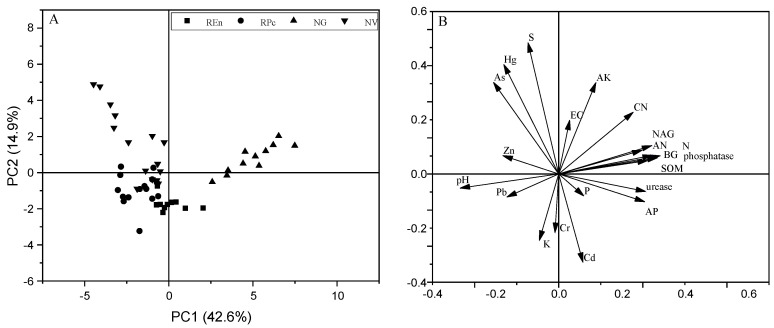
Biplot of the first and second axes obtained from the principal component analysis showing dependencies between soil quality indicators. Principal component analysis scores (**A**) and loadings (**B**).

Principal component analysis (PCA) showed that the first principal component (PC1) accounted for 42.6% of the total variance, while the first two principal component combined accounted for 57% of the total variance (Figure 4). The samples for the physicochemical properties of soil were roughly grouped by the vegetation cover, probably clustering into three differentiated groups. The first group simply contained the samples from NV; the second group was made up of REn and RPc samples; and the last one was represented by NG samples. The soil enzyme activity, SOM, TN, and AN had positive activity with PC1, whereas negative correlated with pH and trace metals.

## 4. Discussion 

Mining activities severely damage the original geological formations and ecosystems, causing inevitable damage to the soil. In this study, the soils from mining areas were characterized by high pH, poor soil quality, and high level of trace metals contamination.

Soil pH moderates the availability of plant nutrients and trace metals mobilization during the process of mine soils restoration [37]. In this study, soil pH was clearly improved after disturbance, as previous studies have also reported [38,39]. Changes in pH of the reclaimed mine soils are generally influenced by bed rock type and the overburden materials. Therefore, the increase in pH may be caused by contamination with unweathered overburden materials containing a significant amount of carbonates [7]. Within a certain concentration range, the salt content of the soil solution is positively correlated with EC. Previous research had observed that EC values in the reclaimed mine soils were higher than those of undisturbed sites [7]. In this study, no significant increase was observed in the disturbed sites, and on the contrary, a significant decrease EC values were observed in REn and RPc, maybe because the sampling time is the rainy season. 

Organic matter and nitrogen are key indicators of soil health, and usually deficient in mine soils, limiting vegetation establishment and sustained productivity [40]. Previously, some scholars have reported that SOM and TN significantly decreased after disturbance, and increased significantly after 5 years of plant restoration in gold mine tailings dams in Central China [41], open-pit phosphate mining in Yunnan Province, China [42], and lignite mining in the northwest of the Czech Republic [43]. In this study, SOM and TN were decreased following mining, and both the SOM and TN content in REn was significantly increased compared with RPc and NV. These results indicate that revegetation by grass accumulates nutrients quicker than woody plants in initial stage of reclamation. Therefore, when selecting woody plants for restoration in this region one should select species with more litter and easy decomposition. Mine soils are generally low in P and K and there is generally no significant change in the P and K content after restoration [43]. Unlike previous studies, a significant increase of P and K content was observed in REn and RPc, which might attributable to the characteristics of the parent rock and weathering of rock materials during the mining process. 

The soil solution nutrient pool consists of nutrients that are dissolved in the water that occupies the pore space within soils and this is where plants and other organisms acquire soluble nutrients [44]. Previous studies have shown that the content of available nutrients does not change significantly in the early restoration stage, even after 20 years of recovery [42,45]. In this study, available nutrients were significantly decrease after disturbance, and AN, AP, AK were not significantly improvement when compared RPc and REn with NV after five years of restoration. This was mainly due to large amount of available nutrient lost during vegetation removal and depletion of SOM. 

Higher extracellular enzymes activity is associated with regions of high nutrient turnover and high primary productivity [46]. Recently, some studies have been demonstrated that metal(loid)s have an adverse influence on the activity of soil enzymes [14,47,48]. Pajak [14] demonstrated that heavy metal pollution particularly reduced activities of urease, and enzyme activity negatively correlated especially with the content of Pb and Cd in the zinc-lead spoil heap soils. Studies about reclaimed mine soils showed remediated soils reached higher enzyme activities than non-remediated areas, with urease and BG showing the greatest discrimination [49]. Monoculture of tree or herb and tree-herb intercropping can increase enzyme activities in metal(loid)-contaminated soil [50]. In this study, after restoration urease, phosphatase and β-glucosidase had slightly increased. However, the soil enzymes activity of mining area soils were significantly lower than that of native grassland, which may be due to high pH, low vegetation coverage and high levels of heavy metal contamination inhibit the source and activity of the soil enzymes. 

The soil trace metals background values in the north Qinghai-Tibetan Plateau was As (7.2 mg kg^−1^), Cd (0.137 mg kg^−1^), Cr (70.1 mg kg^−1^), Hg (0.026 mg kg^−1^), Pb (20.9 mg kg^−1^), Zn (80.3 mg kg^−1^), CN (there is no former research about CN background value in this region, and China Environmental Quality Standard for Soils Grade II requires a CN content < 1 mg kg^−1^) [35,36,51]. In the process of mining and beneficiation, the minerals are usually broken, so it is easy to change the trace metals form and release them into the surrounding environment [12]. Trace metals can affect biological processes and cell components in several ways, for example, by decreasing the respiration of the plant rhizosphere, water, and nutrient uptake, and inhibiting the mitosis of cells [52]. Regarding the mobility of trace metals in this research, Cd, Pb, and Zn with higher mobility, by contrast As, Cr, and Hg had relatively lower mobility [49]. 

As is an element with both metallic and metalloid properties, even at very low concentrations, which could still cause various health problems to plants and animals when ingested continuously for a prolonged period of time, an effect generally referred to as chronic toxicity [12]. High concentrations of As were found in RPc and NV, with values that were 18- and 22-fold greater than the Chinese threshold (25 mg kg^−1^, GB 15618/2018), respectively. Because no As concentration exceeding the limit was observed in REn sites, it can be inferred that maybe As leakage occurred during the mining process in some parts of the mining area. In addition, previous research indicates that as conditions become more acidic and alkaline, As solubility increases, with strong correlations being observed between As leaching with pH in naturally contaminated lands [12,53]. Therefore, the higher soil pH of the disturbed lands may contribute to a continuous release of As. Obviously, the extent of an As-contaminated soil needs to be accurately determined to help recover the contaminated land. In addition to As, the concentration of other trace metals in this study are lower than the Chinese Environmental Quality Standards limit values. Previously, many studies have shown that the content of Cd and Hg in this region has been increasing rapidly in recent years [36,54], but the concentration of both elements in this study was still at a low level. 

## 5. Conclusions

This study determined the mining activities and revegetation effects on soil nutrients, enzymes activities and trace metals. After mining, the pH of mine soils significantly increases and the pH value was negatively correlated with soil nutrients and enzyme activities; and thus, tolerant grass species should be established in such an alkaline soil. The SOM and TN accumulation rate in soils under revegetation by herbage was greater than that in soil revegetation by woody plants after five years of restoration. Soil As content in some areas of the mining region seriously exceeded the statutory limit value. The mechanism whereby As content increased, and As-contaminated land area should be accurately determined, and As-contaminated land needs timely treatment to prevent As from spreading.

## Figures and Tables

**Table 1:** Soil properties and CN content (mean ± standard error) in four sites.

	NG	REn	RPc	NV
pH	7.36 ± 0.10 a	8.26 ± 0.03 a	8.58 ± 0.02 a	8.61 ± 0.06 a
EC (mS m^−1^)	234.55 ± 10.20 a,b	170.90 ± 4.63 b	208.60 ± 9.31 a,b	260.47 ± 26.43 a
SOM (g kg^−1^)	94.46 ± 7.59 a	32.97 ± 3.02 b	16.85 ± 1.89 c	18.99 ± 3.80 c
N (g kg^−1^)	3.38 ± 0.21 a	1.47 ± 0.12 b	1.10 ± 0.06 b,c	0.99 ± 0.14 c
P (mg kg^−1^)	850.1 ± 61.2 b	1031.6 ± 38.6 a	811.7 ± 9.2 b	800.6 ± 101.0 b
K (g kg^−1^)	20.29 ± 0.35 b	22.65 ± 0.17 a	22.04 ± 0.41 a	19.68 ± 0.35 b
S (%)	0.057 ± 0.005 b	0.023 ± 0.002 b	0.018 ± 0.001 b	0.167 ± 0.037 a
AN (mg kg^−1^)	283.85 ± 34.84 a	115.55 ± 10.33 b	106.51 ± 15.38 b	78.61 ± 20.35 b
AP (mg kg^−1^)	464.8 ± 34.76 a	304.2 ± 30.43 b	266.1 ± 12.64 b	324.3 ± 38.52 b
AK (mg kg^−1^)	28.85 ± 1.82 a	26.51 ± 3.14 a	6.65 ± 0.49 b	7.31 ± 1.40 b
CN (mg kg^−1^)	0.088 ± 0.005 a	0.050 ± 0.029 b	0.027 ± 0.003 c	0.056 ± 0.008 b

Different letters (a, b, c) in a line indicate significant differences between the four sites (Tukey’s test, *p* < 0.05). NG = native grassland; REn = restoration by *Elymus nutans*; RPc = restoration by *Picea crassifolia*; NV = nonvegetated; EC = electric conductivity; SOM = soil organic matter; N = total nitrogen; P = total phosphorus; K = total potassium; AN = available nitrogen; AP = available phosphorus; AK = available potassium; CN = cyanide. Same as follows.
